# The function and evolution of the restriction factor viperin in primates was not driven by lentiviruses

**DOI:** 10.1186/1742-4690-9-55

**Published:** 2012-06-26

**Authors:** Efrem S Lim, Lily I Wu, Harmit S Malik, Michael Emerman

**Affiliations:** 1 Department of Microbiology, Fred Hutchinson Cancer Research Center, University of Washington, Seattle, WA, USA; 2 Division of Human Biology, Fred Hutchinson Cancer Research Center, 1100 Fairview Ave. N, P.O., Box 10924, Seattle, WA, 8109-1024, USA; 3 Division of Basic Sciences, Fred Hutchinson Cancer Research Center, Seattle, WA, USA; 4 Howard Hughes Medical Institute, Fred Hutchinson Cancer Research Center, Seattle, WA, USA; 5 Present address: Department of Molecular Microbiology and Pathology & Immunology, Washington University School of Medicine, St. Louis, MO, 63110, USA

## Abstract

**Background:**

Viperin, also known as *RSAD2*, is an interferon-inducible protein that potently restricts a broad range of different viruses such as influenza, hepatitis C virus, human cytomegalovirus and West Nile virus. Viperin is thought to affect virus budding by modification of the lipid environment within the cell. Since HIV-1 and other retroviruses depend on lipid domains of the host cell for budding and infectivity, we investigated the possibility that Viperin also restricts human immunodeficiency virus and other retroviruses.

**Results:**

Like other host restriction factors that have a broad antiviral range, we find that *viperin* has also been evolving under positive selection in primates. The pattern of positive selection is indicative of Viperin's escape from multiple viral antagonists over the course of primate evolution. Furthermore, we find that Viperin is interferon-induced in HIV primary target cells. We show that exogenous expression of Viperin restricts the LAI strain of HIV-1 at the stage of virus release from the cell. Nonetheless, the effect of Viperin restriction is highly strain-specific and does not affect most HIV-1 strains or other retroviruses tested. Moreover, knockdown of endogenous Viperin in a lymphocytic cell line did not significantly affect the spreading infection of HIV-1.

**Conclusion:**

Despite positive selection having acted on Viperin throughout primate evolution, our findings indicate that Viperin is not a major restriction factor against HIV-1 and other retroviruses. Therefore, other viral lineages are likely responsible for the evolutionary signatures of positive selection in *viperin* among primates.

## Background

Antiviral proteins engaged in virus-host interactions are often locked in evolutionary "arms-races", which have been referred to as "Red Queen” conflicts. Viral infections continuously exert immense selective pressures on the host antiviral proteins to evolve adaptively. The signatures of these evolutionary conflicts can be inferred by observing signals of adaptive evolution (also called positive selection) in antiviral genes that result from repeated episodes of Darwinian selection due to past viral infections [1]. Often, the exact amino acids under positive selection can describe the sites and domains involved in host-virus interaction [2-4]. Thus, a detailed look at the evolutionary trajectory of an antiviral gene can provide valuable information about the viral pressures that shaped host evolution.

Viperin (Virus inhibitory protein, endoplasmic reticulum-associated, interferon-inducible, also known as *RSAD2*) is a host protein with broad antiviral activity (reviewed in [5-7]). Viperin inhibits the release of a wide range of viruses in cell culture including Influenza A virus [8], Hepatitis C virus [9-11], and Japanese Encephalitis virus [12]. Moreover, *viperin* knockout mice demonstrate the importance of this protein in controlling West Nile Virus pathogenesis *in vivo*[13]. In the case of human cytomegalovirus (HCMV), Viperin has been reported not only to inhibit the expression of late viral gene products [14] but also to enhance HCMV infectivity by remodeling the cellular actin cytoskeleton [15].

The precise mechanism of the broad-spectrum antiviral function of Viperin remains unclear. However, one model for Viperin antiviral activity links lipid raft disruption to the restriction of Influenza virus release [16]. Lipid rafts are sphingolipid- and cholesterol-enriched microdomains on the plasma membrane that have also been implicated in a number of processes including membrane signaling, polarization, and immunological synapse function [17,18]. Additionally, lipid rafts also play an important role in the entry and assembly stages of viral replication [17,19]. Moreover, the host sterol biosynthesis pathway is downregulated in response to viral infections as part of the innate immune response via type I interferon signaling [20]. Viperin has also been shown to directly inhibit farnesyl diphosphate synthethase (FPPS), a cellular enzyme critically involved in the biosynthesis of isoprenoid-derived lipids [16]. This suggests that the disruption of cellular lipid raft formation may represent a generalized host defense against viruses. As lipid rafts are thought to be sites of assembly and budding for HIV and other retroviruses [17,21-23], we investigated whether Viperin restricts HIV-1 and other retroviruses.

We find that *viperin*, like other host restriction factors against viruses, has evolved under positive selection in primates. We find that Viperin inhibits the release of the LAI strain of HIV-1. However, we show that HIV-1 and SIV strains have intrinsic differences in their sensitivity to Viperin, and most are unaffected by over-expression of Viperin. Furthermore, we did not see an effect of Viperin knockdown on HIV-1 growth. Collectively, our findings suggest that Viperin is not a major restriction factor against HIV-1 and retroviruses, and thus its positive selection must have been driven by other viral pathogens.

## Results

### Viperin has been evolving under positive selection in primates

**Figure 1:**
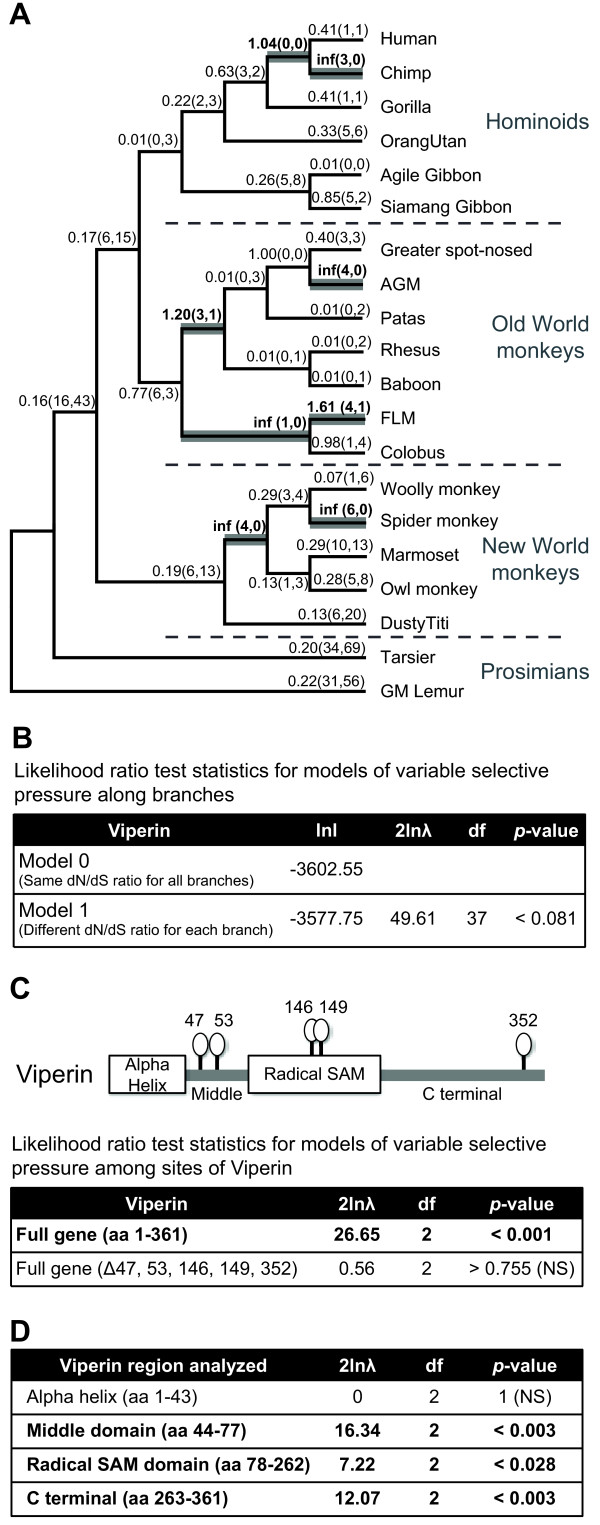
** (A) Cladogram of 20 primate *viperin *genes sequenced for the evolutionary analyses.** Free ratio analysis in PAML was used to calculate the ω (dN/dS) ratios of individual branches. The corresponding ω ratios are shown above each branch, and the number of non-synonymous changes and synonymous changes are indicated in parentheses. Branches with ω > 1 are highlighted in bold. In the case of no observed synonymous changes, the ω ratio could not be calculated (indicated by 'inf'). (**B**) Likelihood ratio test statistics for models of variable selective pressures along branches of primate * viperin *genes are shown, using a comparison between M0 (same dN/dS ratio for all branches) and M1 (different dN/dS ratio for each branch, free-ratio). (**C**) A schematic of Viperin protein domain structure is shown. Residues under positive selection with high confidence (P > 0.95) are indicated in symbols above the protein. The table summarizes the likelihood ratio test statistics for models of variable selective pressure among *viperin* sites (M7 vs M8). Similar results were obtained in a comparison of M1 (neutral) versus M2 (selection) (data not shown). The amino acid positions are annotated in reference to the human Viperin sequence. (**D**) The table summarizes likelihood ratio test statistics performed between the M7 (neutral) and M8 (selection) models for the individual protein domains of *viperin* gene from 20 primate species.

A recurring theme of host restriction factors is that they exhibit a strong signature of positive selection [24]. Given the remarkable breadth of viruses restricted by Viperin [5,6,14,16], we hypothesized that *viperin* might also be evolving under positive selection. To investigate this possibility, we sequenced the *viperin* gene from 18 species of primates and obtained 2 sequences of prosimian *viperin* from Genbank (Figure 1A andAdditional file 1: Figure S1). Together, these primate species span around 60 million years of divergence. The phylogeny constructed from the primate *viperin* sequences was congruent with the generally accepted primate phylogeny [25] confirming that the sequences are orthologous. There was no evidence of recombination as ascertained by a GARD analysis [26].

In order to determine the lineage-specific pressures on the primate *viperin* gene, we performed a free-ratio analysis using the PAML program suite [27], which allows an independent assignment of omega (dN/dS) ratios to each evolutionary branch of the primate phylogeny, where dN/dS ratios > 1 are indicative of positive selection. Several branches of the phylogeny within the New World monkeys, Old World monkeys, and hominoids showed dN/dS ratios > 1 (Figure 1A, bold branches). For instance, the branches leading up to Spider Monkey and FLM have dN/dS ratios > 1, indicative of positive selection. To test whether Viperin was subject to episodic or constant selective pressures over primate evolution, we compared the likelihood ratios of the free-ratio model (Figure 1B, Model 1) where all branches were allowed to have their own independent dN/dS, versus a model where the entire phylogeny had the same dN/dS value (Figure 1B, Model 0). We found that the free-ratio model fit the data better although this was marginally significant (p = 0.08). We therefore conclude that primate *viperin* has been under ancient, episodic positive selection.

We also performed a maximum likelihood analysis using codeml from the PAML program suite [27] that allows for different dN/dS ratios across individual codons, and found strong evidence that the *viperin* gene has been evolving under positive selection in primates (Figure 1C). In order to determine which domain(s) in Viperin are responsible for the signal of positive selection, we examined each domain separately (the N-terminal alpha helix domain, a short middle region, the Radical S-adenosylmethionine (SAM) domain and a flexible C-terminal domain (Figure 1D)). While the N-terminal alpha helix was not under positive selection, the middle region, Radical SAM domain and C-terminal flexible domain showed signs of positive selection with high confidence (Figure 1D). In particular, five amino acid positions exhibit strong signals of positive selection (corresponding to residues 42, 51, 142,145, 352 in human Viperin). These five amino acid residues were independently confirmed to be under positive selection with strong significance by random-effect likelihood (REL) analyses (data not shown) [28]. Importantly, removal of these five amino acids leads to loss of the signature of positive selection from the analyses (Figure 1C), validating that the majority of the positive selection was acting on these sites. The dispersed nature of these positively selected residues is reminiscent of other broadly acting antiviral genes like Protein Kinase R (PKR), wherein escape from viral antagonism drives the positive selection of PKR [29]. This is in contrast to other restriction factors like TRIM5alpha, where a cluster of positive selectively selected residues identifies the viral specificity domains [4]. Therefore, we conclude that *viperin* has evolved under positive selection, likely to escape viral antagonism by a variety of viral lineages over the course of primate evolution.

### Viperin inhibits HIV-1 Lai virus release

**Figure 2:**
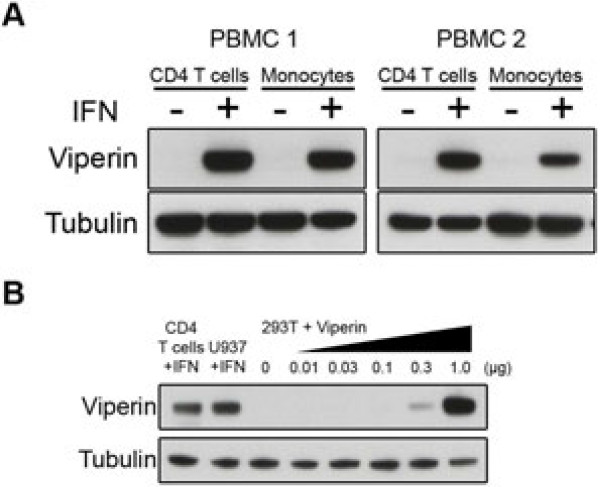
** (A) Viperin expression in HIV-1 primary target cells was determined by Western blot analysis.** CD4+ T cells and monocytes isolated from two donor-derived peripheral blood mononuclear cells were treated with or without interferon β1b induction (500 IU/ml) for twenty hours. (**B**) Western blot analysis of endogenous Viperin expression in primary CD4+ T cells and U937 cells treated with interferon β1b (500 IU/ml) was compared to the transient expression of Viperin (3-fold serial dilutions: 0, 0.01, 0.03, 0.1, 0.3, 1.0 μg) transfected in 293T cells.

Given the broad antiviral range of Viperin, we wished to investigate whether Viperin might also be relevant to restricting HIV-1 infection. We first studied whether Viperin is expressed at the protein level in HIV-1 target cells. Primary CD4+ T cells and monocytes were isolated from peripheral blood mononuclear cells of two donors and treated with interferon β for twenty hours. We found that both primary CD4+ T cells and monocytes express endogenous Viperin after induction with interferon (Figure 2A), but expression levels were undetectable in the absence of interferon.

Given that Viperin is under positive selection and expressed in HIV-1 primary target cells after interferon induction, we investigated whether Viperin restricts HIV-1. To begin these studies, we first compared levels of endogenous Viperin expression with levels achieved by transfection of the cloned human *viperin* gene into 293T cells. We found that untransfected 293T cells express undetectable levels of endogenous Viperin. However, the transient expression of Viperin in 293T cells transfected with between 0.3 and 1 μg of DNA bracketed the amount of endogenous Viperin expression in primary CD4+ T cells and U937 cells when induced with interferon (Figure 2B). Therefore, in subsequent studies, we used amounts of the plasmid encoding the human *viperin* gene that gave levels of Viperin expression just below and just above the levels expressed in primary cells.

**Figure 3:**
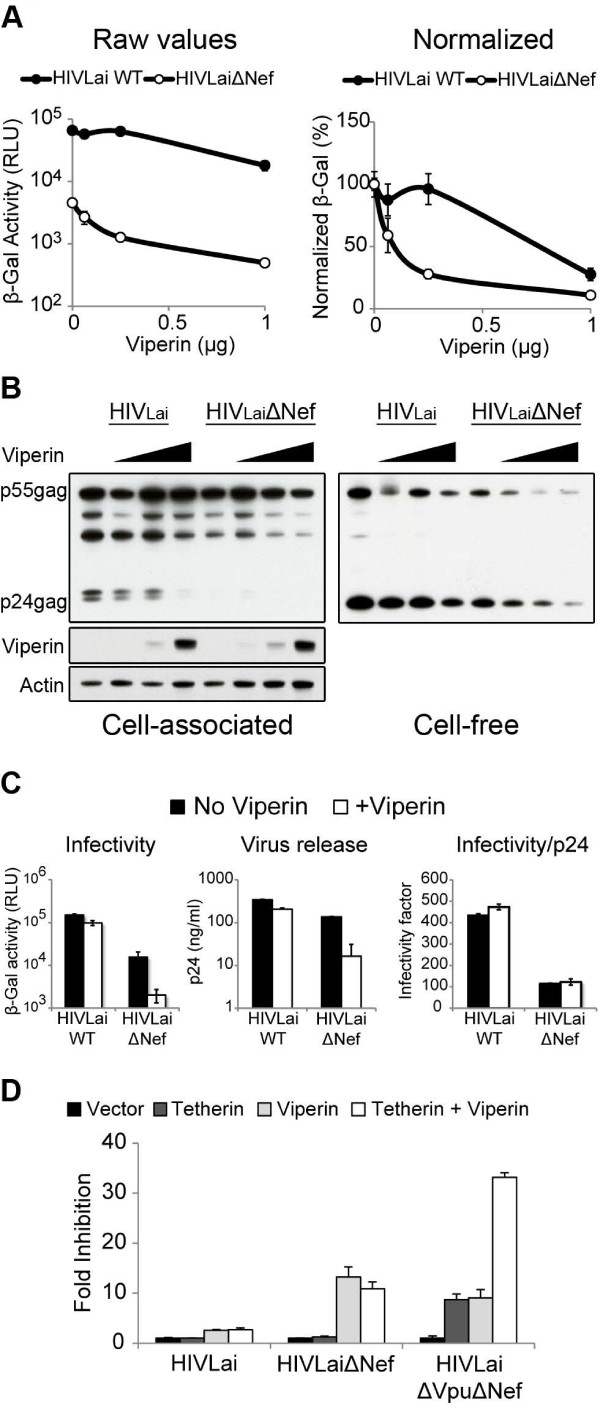
** (A) The effect of viperin was measured by the infectious virus yield.** 293T cells co-transfected with 200 ng of HIVLai or HIVLaiΔNef with serial dilutions of human viperin was titered by infecting TZM.BL indicator cells. The infectivity readout by β-galactosidase activity measured in relative light units (RLU) is shown on the left, and the respective viruses were normalized to β-galactosidase activity in the absence of viperin as shown on the right. Error bars indicate standard deviations from three infection replicates; this data are representative of five independent experiments. (**B**) Western blot analysis was performed on the cell-free virus and cellular extracts, and probed with α-p24 antibody. Viperin expression in the cellular extracts is shown, and actin was probed as a loading control. This blot is representative of four independent experiments. (**C**) The effect of Viperin on the specific infectivity of virus particles was calculated. HIVLai or HIVLaiΔNef virus from 293T cells co-transfected with or without Viperin (700 ng) was titered by infecting TZM.BL indicator cells as shown on the left. Virus release in the cell-free supernatant was quantified by p24 ELISA as shown in the middle. The specific infectivity was calculated as ratio of β-galactosidase activity (RLU) over the amount of p24 (ng/ml), as shown on the right. Error bars indicate standard deviations of triplicate infections; the data are representative of at least three independent experiments. (**D**) Virus yield from 293T cells co-transfected with a combination of Tetherin (50 ng) or Viperin (700 ng) was titered on TZM.BL cells. Fold inhibition was calculated in comparison to virus yield in absence of Tetherin/Viperin. Error bars indicate standard deviations of triplicate infections; the data are representative of three independent experiments.

We tested whether exogenous Viperin expression could restrict HIV-1 by co-transfecting 293T cells with a full-length HIV-1 Lai strain with increasing amounts of the human *viperin* gene. Additionally, we tested HIV-1 Lai lacking a *nef* gene, since Nef has been implicated in modulating cellular cholesterol levels [30,31]. We measured the antiviral activity of Viperin by infecting TZM.BL indicator cells with released virus, and assaying for β-galactosidase reporter activity (See Methods). We found that wild-type HIV-1 virus was marginally affected at low amounts of Viperin, but was inhibited at the highest dose of Viperin (Figure 3A, closed circles). Consistent with the known defect on virion infectivity in the absence of Nef [32], the HIVΔNef virus had a lower infectivity even in the absence of Viperin as measured by the β-galactosidase activity (Figure 3A left, compare closed circles and open circles at 0 μg viperin). Despite that initial observation, the HIVΔNef virus was restricted further by viperin in a dose-dependent manner (Figure 3A, open circles). To compare the degree of restriction between the two viruses, we normalized the β-galactosidase reporter activity of each virus to their measurements in the absence of Viperin (Figure 3A right). We observed that the wildtype HIV virus was only restricted at the highest levels of Viperin expression, whereas the HIVΔNef virus was more sensitive to Viperin restriction than wildtype HIV, even at the lower levels of Viperin expression.

Because Viperin restricts influenza virus at the step of virus release [16] and HCMV by inhibiting the production of viral structural proteins [14], we investigated whether HIV-1 production and/or release is affected by Viperin by Western blotting for cell-associated and cell-free Gag proteins. We hypothesized that if Viperin affects HIV production; we expected to see a decrease in intracellular p55gag expression that correlates with a decrease in cell-free p24gag. Conversely, if Viperin affects virus release, we would see lower levels of cell-free p24gag while levels of p55gag would remain unchanged.

We found that cell-associated HIV-1 p55gag for both WT and ΔNef virus was only marginally affected by the expression of Viperin (Figure 3B). Moreover, cell-free levels of p24gag from wild type HIV-1 were modestly affected by the expression of Viperin (Figure 3B) in a manner consistent with a slight decrease in the amount of supernatant HIV p24gag when measured with an enzyme linked immunosorbent assay (ELISA) assay (Figure 3C, middle). However, Viperin expression showed a drastic reduction in cell-free HIVΔNef p24gag (Figure 3B, right), with only a small effect on intracellular p55gag levels (Figure 3B, left). This suggests that Viperin affects release of HIVΔNef virus.

Since Viperin might also be affecting the quality of the virus particles, we quantified the specific infectivity of virus particles by measuring the ratio of infectious titer to relative particle production (by p24 ELISA). Consistent with other studies, we found that wildtype HIV virus was more infectious than HIVΔNef virus (Figure 3C). However, viperin expression did not affect the specific infectivity (infectivity divided by p24gag) of either wildtype HIV virus or HIVΔNef virus particles (Figure 3C, right), indicating that the Viperin-mediated restriction of HIV-1 is not due to a reduction in viral infectivity.

Since Viperin seemed to affect virus release, we compared Viperin restriction to that of Tetherin, a well-characterized host restriction factor that inhibits virus release [33,34]. Virus restriction by a combination of Viperin and Tetherin expression was roughly additive (Figure 3D). Furthermore, the response of Viperin and Tetherin is different since HIV-1 Vpu abrogates Tetherin restriction but has no effect on Viperin restriction, whereas HIV-1 Nef abrogates Viperin restriction (Figure 3D). These results suggest that Viperin restricts HIV-1 release by a mechanism that is distinct from the pathway used by Tetherin.

### Most HIV strains, SIVs and retroviruses are resistant to Viperin restriction

**Figure 4:**
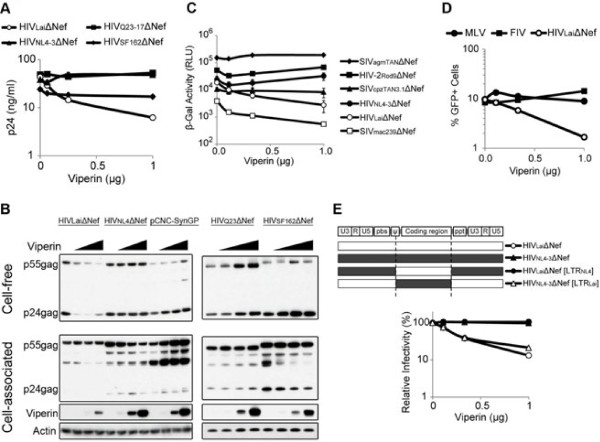
** A panel of HIV-1ΔNef strains was tested for their sensitivity to Viperin overexpression.** (**A**) Virus release was assayed by p24 ELISA quantification of cell-free supernatant forty-eight hours after co-transfection of 293T cells. Error bars indicate standard deviations of three replicates and are representative of three independent experiments. (**B**) Western blot analysis was performed on the cell-free virus and cellular extracts of (A). HIV-1 Gag expression was assessed by α-p24 antibody. Viperin expression in the cellular extracts is shown, and Actin was probed as a loading control. This blot is representative of at least three independent experiments. (**C**) Single-cycle infectious virus yield of VSV-G pseudotyped primate lentiviruses from 293T cells co-transfected with serial titration of Viperin and was titered on TZM.BL indicator cells. Proviruses were deleted of the *nef* gene. The infectivity readout by β-galactosidase activity was measured in relative light units (RLU). (**D**) The effect of Viperin on retroviruses was measured by co-transfecting 293T cells with MLV, FIV or HIVLaiΔNef with serial titrations of Viperin. Viruses were pseudotyped with VSV-G and titered by infecting HeLa cells. The expression of virus-encoded GFP was quantified by flow cytometry. This analysis is representative of at least three independent experiments. (**E**) Schematic of chimeric proviruses of HIVLai and HIVNL4-3 highlighting the breakpoint and features in the non-coding region of the provirus. Single-cycle infectious virus yields from co-transfected 293T cells were assayed by titering on TZM.bl reporter cells. Error bars indicate standard deviations of four infection replicates, and the data are representative of at least three independent experiments.

To examine the breadth of Viperin restriction on HIV-1 we tested several strains of HIV-1 for their susceptibility to Viperin restriction on virus release. The proviruses were deleted of their *nef* gene to exclude the possible confounding effects of Nef specificity. Consistent with the earlier experiments (Figure 3C), HIVLaiΔNef virus release was inhibited by human Viperin. However, virus release of the HIVΔNef NL4-3, SF162 (both HIV-1 subtype B) and Q23-17 strains (HIV-1 subtype A) were unaffected by Viperin expression (Figure 4A). To verify these observations, we performed a Western blot analysis comparing the cell-associated and cell-free HIV-1 Gag protein levels. In contrast to the dose-dependent inhibition of HIVLaiΔNef virus release, cell-free HIVNL4-3ΔNef virus release remained unaffected (Figure 4B). While there was an observable effect on intracellular HIVNL4-3ΔNef virus p55 levels, this difference was not reflected in the cell-free Gag p24 levels or the ELISA assay. In addition, we tested a widely used HIV-1 vector encoded from a codon-optimized Gag-pol sequence called pCNC-SynGP [35]. We observed that the cell-free HIV-1 pCNC-SynGP Gag was unaffected by Viperin expression. Instead, cell-associated p55gag protein production was slightly increased in the presence of Viperin expression. As for the HIVΔNef SF162 and Q23-17 strains, there were no significant effects on cell-free p24gag or cell-associated p55gag expression levels. One exception is a noticeable decrease in the partially processed, cell-associated, HIVΔNef SF162 p40gag levels. However, while Viperin might have a subtle effect on the intracellular Gag levels of certain HIVΔNef strains, the difference was not reflected in the cell-free virus or measured in the ELISA assay. Thus, it appears that Viperin does not significantly impact the virus release of most HIVΔNef strains tested except for the HIV-1 Lai strain.

We next investigated the ability of Viperin to restrict related simian immunodeficiency virus (SIV). In addition to their *nef* gene deletion, the proviruses were also pseudotyped with VSV-G so that the entry of all viruses would be equal. Using an infectivity assay, we found that SIVmac239ΔNef was as sensitive to Viperin as HIV-1LaiΔNef (Figure 4C, open squares). However, SIVagmTAN1ΔNef, SIVcpzTAN3.1ΔNef and HIV-2ROD9ΔNef were resistant to Viperin restriction. Since Viperin did not appear to restrict the majority of primate lentiviruses we tested, we also examined two additional divergent retroviruses – murine leukemia virus (MLV) and feline immunodeficiency virus (FIV). In contrast to the control HIVΔNef Lai virus, MLV and FIV were unaffected by Viperin expression (Figure 4D), indicating that Viperin does not generally restrict retroviruses. Thus, while Viperin may inhibit a limited subset of primate lentivirus strains (HIV-1Lai and SIVmac239, for example), the majority of HIV-1 strains, SIVs and retroviruses that we tested are not affected by Viperin expression.

The difference in restriction profiles between Lai and NL4-3 strains was unexpected since NL4-3 is a recombinant virus of NY5 and Lai strains [36]. We attempted to map the viral determinant of Viperin sensitivity by constructing a series of chimeric proviruses between Lai and NL4-3 strains and tested them in an infectivity assay. While we expected that changes in Gag proteins previously associated with virus release might be involved, chimeric proviruses within Gag failed to identify a determinant within Gag (data not shown). Instead, we found that even when all coding regions were swapped between the Lai and NL4-3 strains of HIV-1, the sensitivity to Viperin restriction still mapped to Lai sequences outside of the coding region (Figure 4E). That is, when the coding region of HIVLai was expressed in the context of HIV-1 NL4-3 non-coding region sensitivity to Viperin restriction was lost. However, when we inserted the non-coding region of HIV-1 Lai including the LTRs, 5’ packaging region, and the PPT into HIV-1 NL4-3, then the virus was sensitive to inhibition by Viperin (Figure 4E). Thus, human Viperin displays differential restriction specificities against related HIV-1, which is dictated entirely by non-coding regions of the provirus. We conclude, therefore, that the restriction by exogenous Viperin of HIV-1 is likely due to threshold effects of expression, rather than due to direct interactions of Viperin with viral components.

### Endogenous viperin does not inhibit HIV-1 Lai

**Figure 5:**
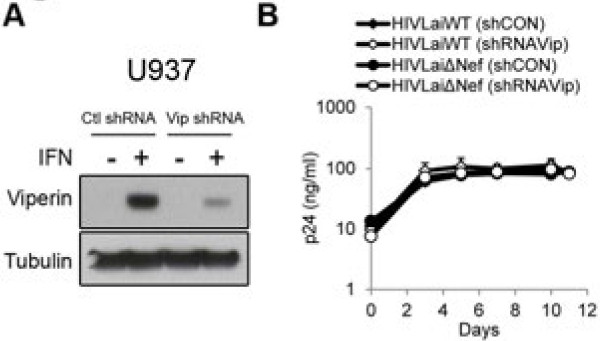
** Analyses of endogenous Viperin activity.** (**A**) Viperin expression in U937 cells stably transduced with empty pGIPZ shRNA vector (Ctl) or Viperin targeting shRNA (Vip) was analyzed by Western blot twenty hours after interferon β1b treatment (500 IU/ml). (**B**) Spreading infection of wildtype HIVLai or HIVLaiΔNef was quantified by p24 ELISA at indicated time points after infecting shRNA-transduced U937 cells at an moi of 0.5. Cells were maintained in interferon β1b (500 IU/ml) for the duration of the experiment. The data are representative of at least three independent experiments.

A more critical test of the physiological role of Viperin on HIV-1 replication is to change levels of endogenous Viperin in cells where spreading infections can be performed. To determine the effect of endogenous Viperin on HIV-1, we sought to knock-down Viperin expression with shRNAs. Unfortunately, we were unable to obtain suitable stable or transient knockdown in primary T cells (data not shown). Many T cell lines such as SupT1 cells also do not express Viperin after interferon induction (data not shown). However, CD4+ U937 monocytic cells do express Viperin after interferon induction (Figure 2B). Using a stably transduced shRNA construct, we were able to partially knockdown expression of Viperin in U937 cells (Figure 5A). U937 cells that were either knocked-down for Viperin (shRNAVip) or transduced with a control shRNA (shCON) were infected with HIV-1LAI and HIV-1LAIΔNef at a multiplicity of infection of 0.5. Infections were done in the presence of interferon to induce Viperin expression. Viral supernatant was collected periodically over 11 days, and spreading infection was monitored by p24 ELISA. We found that there was no significant difference between WT and ΔNef virus growth in the cells knocked-down for Viperin expression (Figure 5B). Considering that the HIVLaiΔNef is the most sensitive strain in Viperin overexpression experiments (Figuresa 2 and 4), these results suggest that endogenous levels of Viperin do not affect spreading infection by HIV-1.

### No functional divergence in lentiviral restriction among primate Viperin orthologs

Most of the experiments that we have carried out were using the human *viperin* allele. However, since *viperin* evolves rapidly under positive selection, we might not be accurately capturing the potential ability of Viperin proteins to restrict lentiviruses. The species-specificity of action is one of the key features that have emerged from the study of rapidly evolving restriction factors. To address the possibility that the human Viperin might not accurately capture the restrictive potential of Viperin, we carried out two experiments to measure any functional divergence between primate Viperin orthologs that may have arisen from the positive selection.

**Figure 6:**
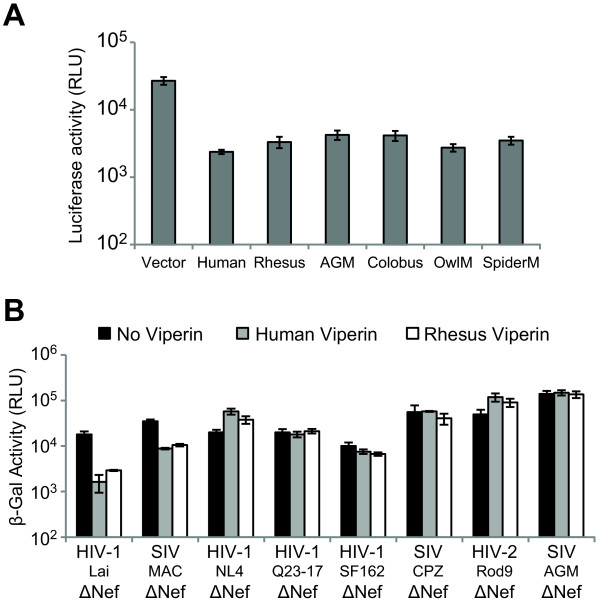
** The antiviral activity of primate Viperin orthologs was assayed.** (**A**) 293T cells were co-transfected with HIVLaiΔNef that encodes a luciferase reporter gene (200 ng) and the indicated primate Viperin (700 ng). Virus yield was measured by infecting SupT1 cells and assayed for luciferase expression. Error bars represent standard deviations of four infection replicates and are representative of three independent experiments. (**B**) Single-cycle infectious virus yield of VSV-G pseudotyped primate lentiviruses from 293T cells co-transfected with 700 ng of human Viperin, rhesus Viperin or empty vector was titered on TZM.BL indicator cells. The infectivity readout by β-galactosidase activity was measured in relative light units (RLU). Error bars indicate standard deviations of four infection replicates, and are representative of at least three independent experiments.

First, we tested 5 additional Viperin orthologs against the HIVLaiΔNef (Figure 6A). We found that all six Viperin orthologs are able to restrict this virus to approximately the same extent, despite some variation in the degree of restriction. This means that the positive selection of *viperin* does not manifest a functional difference in the degree of restriction of HIVLaiΔNef. Second, we compared the human and rhesus orthologs against a panel of viruses to assess whether we could discern any key restriction differences between these two Viperin orthologs (Figure 6B). It is notable that human versus rhesus differences have been found in many positively selected restriction factors that have been tested so far [4,29,37]. However, in the case of Viperin, we found no significant differences between the restriction profiles of human and rhesus Viperin.

These results imply that Viperin's lack of restriction of the majority of lentiviruses and retroviruses tested is not a consequence of testing only one Viperin allele. Moreover, this finding strongly implies that gain or loss of lentivirus restriction is not correlated with the dramatic evolutionary changes we observed in the *viperin* gene in primates.

## Discussion

### Primate viperins are not lentiviral restriction factors

The restriction factor Viperin recognizes and restricts a wide diversity of viruses, including both single-stranded RNA and double-stranded DNA viruses [5-7]. This broad repertoire of antiviral activity prompted us to investigate Viperin’s restrictive activity against retroviruses, specifically the primate lentivirus lineage. We found that Viperin is highly interferon-induced in primary target cells of HIV. Viperin overexpression is able to inhibit HIV-1 Lai replication by affecting virus release. However, most other strains of HIV-1, SIV and other retroviruses are unaffected by primate Viperin orthologs. Finally, endogenous Viperin does not inhibit the spreading infection of HIV-1 Lai. Therefore, we conclude that Viperin is not a major restriction factor against HIV-1 and other primate lentiviruses. These results emphasize the fact that broadly acting innate host defense genes do, nonetheless, have viral specificity that goes beyond their escape from viral antagonism.

Lipid rafts play an important role in virus replication and are actively regulated as part of the host response to viral infection [20]. As Viperin inhibits Influenza virus release by impairing the lipid metabolic pathway enzyme FPPS resulting in the disruption of lipid rafts [16], we expected that it would have a broad antiviral role in inhibited viruses that bud through lipid rafts, in a way similar to how Tetherin affects many different enveloped viruses that bud through the plasma membrane [38]. However, importantly, we did not find this to be the case since HIV was generally resistant to the effects of Viperin. Thus, these data argue that it is overly simplistic to characterize all viral lipid raft interactions as equivalent, but rather there are likely important differences in the lipid requirements for budding of different virus families. It may be important that unlike HIV, influenza assembly at lipid rafts does not involve the ESCRT machinery [39].

A previous study that showed that poly I:C-induced Viperin had a subtle effect on HIV-1 infection in astrocytes [40]. However, our findings are not consistent with an effect of Viperin on HIV-1 replication in general. Although other retroviral restriction factors appear to be active in all cells tested, it is possible that the antiviral effects of Viperin are cell-specific and would be active in primary cells that were not able to be tested in this study. Furthermore, it is formally possible that all of the retroviruses tested encode an antagonist of Viperin that abrogates its action. However, virus-host antagonism should show species-specificity [1]; we believe that this is very unlikely because *viperin* cloned from a wide range of different primates showed equivalent activities against HIV-1 and diverse retroviruses (Figure 6 and data not shown), and Viperin expression is not affected by co-transfection with proviruses (data not shown). Nonetheless, the fact that we did find two lentiviral proviruses encoded by HIV- 1 Lai and SIVmac239 that were inhibited by transfection of *viperin,* suggests that the pathway used by Viperin must at least peripherally intersect with lentiviral production.

Our findings of Viperin restriction of the Lai strain of HIV-1 but not the NL4-3 strains are unexpected since NL4-3 is a recombinant virus of NY5 and Lai strains [36]. We have mapped the genetic basis of the susceptibility difference to a non-coding region of the virus. Moreover, we found that HIV-1Lai with a deletion in Nef was more sensitive to Viperin than a full-length provirus. Complementation of the Lai strain of HIV-1 ΔNef provirus with Nef *in trans* partially restored the resistance to Viperin (data not shown). One possible explanation is that LTR promoter efficiency and the presence of Nef may affect viral Gag production in a manner that renders it sensitive to Viperin. Alternatively, we believe it is more likely that less infectious viral combinations are more sensitive to perturbations caused by exogenous expression of Viperin. Nonetheless, considering that most strains of HIV-1, SIVs (excluding SIVmac239), tested are resistant to Viperin, we favor the more parsimonious conclusion that Viperin is not a significant player in the immune defense against lentiviruses. Moreover, the results describe here serve as an important caution that over-expression systems with single isolates cannot be relied on to functionally identify and characterize restriction factors.

### Insight into viperin function from its positive selection

Antagonistic genetic conflicts between hosts and viruses have driven rapid adaptive evolution of antiviral protein which is characteristic of many retroviral restriction factors [24] as well as other antiviral factors that target a broad range of viruses [29,41]. Like many host restriction factors, we find that *viperin* has been evolving under positive selection in primates. The signatures of rapid evolution in *viperin* may provide valuable information about the mechanism by which it restricts this broad repertoire of viruses and likely avoids viral antagonism. This is analogous to the dynamics of the host restriction factor Tetherin, where the highest recurrent signal of positive selection corresponds to the amino acid that is a determinant for antagonism by Nef [3]. In the antagonist-driven scenario, we speculate that the amino acid residues under positive selection on Viperin might have been driven by pressures to evade viral antagonists and would be indicative of sites directly involved in viral protein interactions. In this regard, the evolution of primate Viperin may provide valuable clues to virus families that have driven the positive selection of Viperin throughout primate evolution [1,41] by finding which viruses encode antagonists to Viperin with a specificity that is specified by the amino acids in Viperin that are under positive selection. A promising candidate would be Japanese encephalitis virus which encodes an unidentified viral antagonist that degrades Viperin in a proteasome-dependent mechanism [12].

## Conclusion

We document an ancient, episodic and recurrent history of adaptive evolution in Viperin over primate evolution. However, despite the fact that Viperin restricts other a wide range of other virus families [15], it does not have a major effect on HIV-1 and other lentiviruses, and therefore, the positive selection in *viperin* was likely driven by selective pressures imposed by virus families other than the lentiviruses.

## Methods

### Plasmids

Human Viperin was cloned from human cDNA derived from 293T cells, and inserted into a retroviral expression vector pLPCX as an untagged construct. The five primate Viperin orthologs were similarly cloned from cDNA into the pLPCX retroviral expression vector as untagged constructs. HIVLai, HIVLaiΔNef and HIVLaiΔVpuΔNef, SIVagmTANΔEnvΔNef were described previously [3,42]. HIVNL4-3ΔNef was obtained from the NIH AIDS Research and Reference Reagent Program, 11100. HIVSF62ΔNef was generated by fill-on of the XhoI site (nt 8576) resulting in a 2bp frameshift mutation in the Nef open reading frame of the full length HIV-1SF162 provirus [43]. HIV-1Q23-17ΔNef was constructed by introducing a luciferase gene in place of Nef into the full length HIV-1 Q23-17 provirus [44]. SIVcpzΔNef was generated by introducing a luciferase gene in place of Nef into the full length SIVcpzTAN3.1 provirus [45] (NIH AIDS Research and Reference Reagent Program, 11100) by overlapping PCR between the NdeI and NheI region and sequence verified. HIV-2Rod9ΔEnvΔNef was a gift from Masahiro Yamashita, and SIVmac239ΔEnvΔNef was a gift from David Evans [46]. pGIPZ vector-based control shRNA or shRNA targeting Viperin mRNA (hairpin construct: TGCTGTTGACAGTGAGCGCGATGAAAGACTCCTACCTTATTAGTGAAGCCACAGATGTAATAAGGTAGGAGTCTTTCATCTTGCCTACTGCCTCGGA) were purchased from FHCRC RNAi core facility.

### Viral infectivity assay

293T cells were seeded at 1.67 x 10^5^ cells/ml in 12-well plates, and DNA was transfected with TransIT LT-1 (Mirius) according to the manufacturer's recommendations. The total amount of DNA in all transfections was maintained constant with appropriate empty vectors. Forty-eight hours after transfection, supernatant was collected, filtered through a 0.2μM filter and serially diluted for the following infectivity assay. SupT1 cells at 2.5 x 10^5^ cells/ml in 96-well plates were as described previously [3], or TZM.bl cells at 1.0 x 10^5^ cells/ml in 96-well plate were as described previously [42]. The β-Galactosidase activity was detected using the Galacto-Star system (Applied Biosystems) according to the manufacturer's recommendations.

### Virus release p24 ELISA

Virus were serially diluted and measured by HIV-1 p24 antigen capture assay (Advanced BioScience Lab Inc) and detected with QuantaRed enhanced chemifluorescent HRP substrate (Thermo Scientific) according to the manufacturer’s protocol.

### Western blotting

Western blot analysis was performed as described previously [3,42] with the following antibodies: HA-specific antibody (Babco), anti-Viperin (Enzo Life Sciences), anti-actin (Sigma-Aldrich), anti-tubulin (Sigma-Aldrich), and HIV-1 p24 antibody (NIH Aids Research and Reference Reagent Program, 183-H12-5C) [47]. Primary antibodies were detected with a corresponding horseradish peroxidase-conjugated secondary antibody.

### PBMC isolation and separation

Patient pall filters were obtained from Puget Sound Blood Center. PBMCs were isolated by standard ficoll histopaque gradient methods. Monocytes and CD4+ T cells were isolated by Human CD14 selection and CD4+ magnetic bead isolation (EasySep), and the isolation purity (>97-99%) was confirmed by flow cytometry staining (BD Pharmingen). Monocytes were maintained in RPMI containing 10% FBS. CD4+ T cells were activated with 2.5μg/ml PHA and 20U/ml IL-2 for 3 days before interferon treatment. Monocytes and CD4+ T cells were treated with 500 IU/ml human interferon β1b for 20 hours, followed by western blot analysis on total cell lysates.

### Spreading infectivity assay

U937 cells stably transduced with either a Viperin-targeting shRNA or control shRNA constructs were infected with a wild type HIV-1Lai virus or HIV-1LaiΔNef at a moi of 0.5. Cells were washed with PBS three times and maintained in media containing 500 IU/ml human interferon β1b throughout the course of the experiment. Supernatant was collected at indicated time points and virus was quantified by p24 ELISA.

### Sequencing of primate *viperin* genes

The *viperin* genes from the following primates were amplified from RNA isolated from cell lines obtained from Coriell Cell Repositories (Camden, NJ): chimpanzee (*Pan troglodytes*), gorilla (*Gorilla gorilla*), Sumatran orangutan (*Pongo pygmaeus*), Siamang gibbon (*Hylobates syndactylus*), agile gibbon (*Hylobates agilis*), rhesus macaque (*Macaca mulatta*), greater white-nosed monkey (*Cercopithecus nictitans*), kikuyu colobus (*Colobus guereza kikuyuensis*), Francois' leaf monkey (FLM) (*Trachypithecus francoisi*), spider monkey (*Ateles geoffroyi*), owl monkey (*Aotus trivirgatus*), dusty titi monkey (*Callicebus moloch*) and woolly monkey (*Lagothrix lagotricha*). Human (*Homo sapiens*)*,* African green monkey (*Chlorocebus aethiops*) and Baboon (*Papio anubis*) Viperin were amplified by reverse transcription-PCR (RT-PCR) from an RNA extract of 293T cells, COS-7 cells and B-LCL cells respectively. Viperin was amplified by RT-PCR with a OneStep RT-PCR kit (Qiagen), and the cDNA derived was directly sequenced. Viperin was amplified with "forward" primer (5'-ATGTGGGGTGCTTACACCTGCTGCTTTTGCTG-3') or (5'-ATGTGGGTACTCACGCCTGCTGCTTTTGCTG-3') in combination with "reverse" primer (5'-CTACCAATCCAGCTTCAGATCAGCCTTACTC-3') or (5'-CTACCAATCCAGCTTCAGATCAGCCTTACTC-3'). Sequences for prosimian grey mouse lemur (*Microcebus murinus*) and tarsier (*Tarsius syrichta*) *viperin* gene were obtained by tblastx search on the NCBI database from cont1.216710 (ABDC01216711.1) and contig1.93320 (ABRT010093321.1) respectively.

### Sequence analysis

DNA sequences were aligned by ClustalX [48] and were edited manually. The amino acid positions are annotated in reference to the human Viperin sequence. A phylogeny of *viperin* genes was constructed from DNA sequences with ClustalX by the neighbor-joining method using the Jukes Cantor method of correction and with PhyML [49] by the maximum-likelihood method. The two methods yielded trees with identical topologies. Maximum-likelihood analysis was performed with CODEML from the PAML suite of programs [27] as previously described [2,3]. Sequence alignments were obtained when the data were fitted with an F61 model of codon frequency, and consistent results were obtained when the data were fitted with an F3 x 4 model of codon frequency. *Viperin* sequences were fitted to NSsites models that disallowed (NSsites model 1 and 7) or permitted (NSsites model 2 and 8) positive selection. Likelihood ratio tests were performed to evaluate whether permitting codons to evolve under positive selection gave a better fit to the data. A cutoff of posterior probability of p > 0.95 was implemented in these analyses (M8) to identify amino acid residues having evolved under positive selection. Analyses were also validated with REL from the HyPhy package [50]. Free ratio analysis in PAML was used to calculate the ω (dN/dS) ratios of individual branches. Likelihood ratio test statistics was performed for models of variable selective pressures along branches of primate *viperin* genes between M0 (same dN/dS ratio for all branches) and M1 (different dN/dS ratio for each branch). The degree of freedom is equal to one less than the total number of branches in the phylogeny.

### Nucleotide sequence accession numbers

The sequences of the 18 primate *viperin* genes have been entered into the GenBank database under accession numbers NM_080657, JQ437821 to JQ437837.

## Competing interests

The authors declare that they have no competing interests.

## Authors’ contributions

ESL performed all experiments and except for Figure 5 which was done by LIW. ESL, HSM and ME designed experiments, analyzed data, and wrote the manuscript. All authors read and approved the final manuscript.

## Supplementary Material

Additional file 1 **Figure 1. Amino acid alignment of 20 primate viperin genes sequenced for the evolutionary analyses.** The protein domain structure is shown above the alignment, and residues under positive selection are highlighted in bold. The amino acid positions are annotated in reference to the human Viperin sequence. Click here for file
